# How early should we ablate ventricular tachycardia? From rescue to prevention in ischaemic VT

**DOI:** 10.1093/europace/euag180

**Published:** 2026-07-14

**Authors:** Francesco Santoro, Martina Molinari, Pier Luigi Pellegrino, Natale Daniele Brunetti

**Affiliations:** Cardiology Unit, Department of Medical and Surgical Sciences, University of Foggia, Viale Pinto n.1, Foggia 71122, Italy; Cardiology Unit, Department of Medical and Surgical Sciences, University of Foggia, Viale Pinto n.1, Foggia 71122, Italy; Cardiology Unit, Department of Medical and Surgical Sciences, University of Foggia, Viale Pinto n.1, Foggia 71122, Italy; Cardiology Unit, Department of Medical and Surgical Sciences, University of Foggia, Viale Pinto n.1, Foggia 71122, Italy

## Abstract

**Graphical Abstract:**
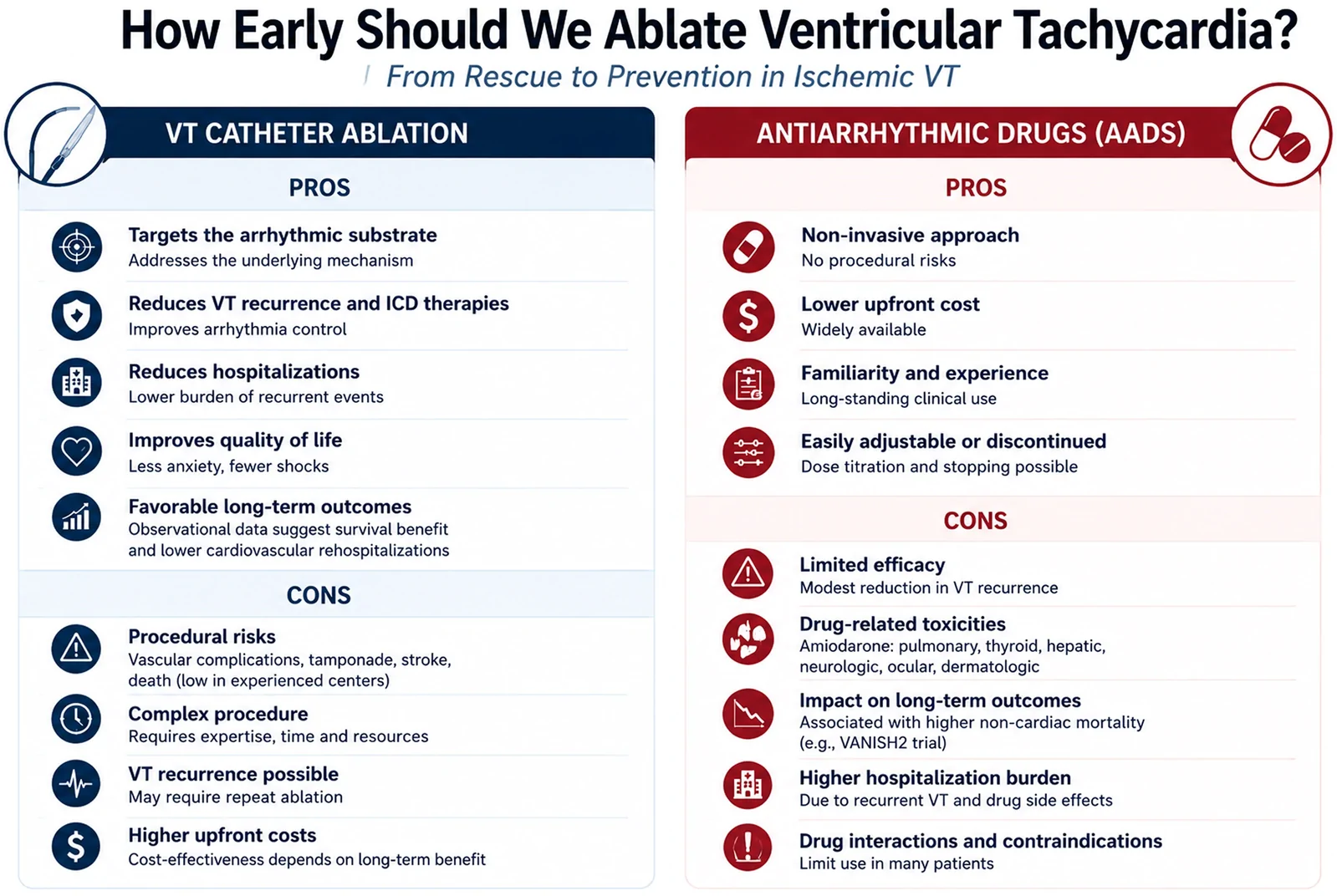
Schematic illustration of the evolving treatment paradigm for ischaemic ventricular tachycardia, highlighting the increasing role of catheter ablation relative to anti-arrhythmic drug therapy based on evidence from recent randomized clinical trials.

Schematic illustration of the evolving treatment paradigm for ischaemic ventricular tachycardia, highlighting the increasing role of catheter ablation relative to anti-arrhythmic drug therapy based on evidence from recent randomized clinical trials.

**This editorial refers to ‘Early catheter ablation versus antiarrhythmic drugs in treatment-naïve ischaemic ventricular tachyarrhythmias: a meta-analysis of randomized controlled trials’, by T. M. Façanha *et al.*, https://doi.org/10.1093/europace/euag073.**

The management of ventricular tachycardia (VT) in patients with ischaemic heart disease has progressively evolved over the last two decades. Initially regarded as a rescue therapy reserved for highly symptomatic patients with recurrent implantable cardioverter-defibrillator (ICD) shocks despite anti-arrhythmic drug (AAD) therapy, catheter ablation (CA) is increasingly being considered earlier in the disease course. The meta-analysis by Façanha *et al*. published in this issue of *Europace* adds another important piece to this evolving puzzle by specifically focusing on treatment-naïve patients with ischaemic ventricular tachyarrhythmias.^1^

Historically, VT ablation developed within a ‘failure-driven’ framework. Patients were generally referred for ablation only after experiencing recurrent ventricular arrhythmias, repeated ICD therapies, ventricular storm, or intolerance to escalating AAD regimens, particularly amiodarone. Therefore, AAD therapy—despite modest efficacy and well-known toxicities—became the first-line therapeutic approach.

However, this traditional paradigm raises an important question: is waiting for recurrent VT episodes, ICD shocks, or chronic drug toxicity truly the optimal strategy for these patients?

Waiting for recurrent ICD shocks before referral may increasingly represent a missed therapeutic opportunity. Over the years, increasing evidence has demonstrated that ICD therapies are not benign events. Appropriate shocks are associated with impaired quality of life, repeated hospitalizations, psychological distress, progressive heart failure, and worse long-term outcomes.^2^ Similarly, long-term exposure to amiodarone carries substantial cumulative toxicity involving pulmonary, thyroid, hepatic, neurologic, and systemic adverse effects. Thus, the clinical burden associated with recurrent ventricular arrhythmias extends far beyond arrhythmia recurrence alone.

In this context, the concept of early substrate modification has progressively emerged. Rather than acting solely as a rescue intervention, CA may potentially alter the clinical trajectory of patients before recurrent arrhythmia burden accumulates. The key issue is therefore no longer whether ablation can suppress VT recurrence, but rather whether earlier intervention can prevent the downstream consequences of recurrent electrical instability. Recent randomized evidence, including PARTITA and MANTRA-VT, further supports the concept that earlier substrate-based ablation may improve disease stability before recurrent arrhythmic burden accumulates.^3,4^

The present meta-analysis addresses this issue by evaluating CA vs. first-line AAD therapy in treatment-naïve ischaemic VT patients. Importantly, these findings extend previous evidence from broader randomized meta-analyses comparing CA and AAD therapy in ischaemic VT populations, which had already demonstrated reductions in ICD therapies, cardiovascular rehospitalizations, and treatment-related adverse events.^5^ Pooling three randomized controlled trials and 618 patients, the authors found no significant differences in all-cause mortality, ICD therapies, ICD shocks, or ventricular storm between strategies. Nevertheless, early CA was associated with numerically lower rates of hospitalizations and adverse events, although these findings were largely driven by the SURVIVE-VT trial.

Importantly, these findings should not be interpreted as evidence against early ablation. Rather, they highlight the complexity of demonstrating mortality benefit in ischaemic cardiomyopathy. Death in this population is frequently driven by progressive heart failure, comorbidity burden, autonomic dysfunction, and non-cardiovascular causes. Moreover, the trials included were not powered for mortality endpoints, that may require larger studies.

At the state of the art, the question may be whether earlier ablation can improve disease stability and reduce cumulative morbidity. From this perspective, the observed reductions in hospitalization burden and treatment-related adverse events become clinically meaningful.

Indeed, the comparison between procedural risk and chronic pharmacological toxicity deserves particular attention. Ventricular tachycardia ablation is still frequently perceived as an invasive procedure with a high risk of in-hospital complication, whereas AAD therapy is often considered the conservative approach. However, the cumulative toxicity of long-term amiodarone exposure may be underrecognized. Recent data from the VANISH2 trial showed that amiodarone exposure was associated with a three-fold increase in non-cardiac death when compared with patient undergoing VT ablation.^6^ On the other side, in high-volume centres, procedural safety of VT ablation has improved considerably owing to advances in electroanatomical mapping,^7^ substrate characterization,^8^ intracardiac imaging,^9^ energy sources,^10,11^ and peri-procedural management.^12^ Therefore, the balance between procedural risk and long-term drug-related harm may no longer favour delayed intervention (*Graphical abstract*).

At the same time, caution remains necessary. The available randomized evidence remains limited, and the treatment effect appears strongly influenced by individual studies, crossover rates, endpoint definitions, and evolving technologies.

This uncertainty underscores another critical aspect: timing alone is probably insufficient. Patient selection is likely to become equally important. Ischaemic VT is not a homogeneous disease. The arrhythmic substrate varies according to scar burden, scar heterogeneity, ventricular function, autonomic profile, inflammatory activity, and extent of structural remodelling. Future management strategies will therefore likely rely on individualized risk stratification rather than fixed stepwise algorithms.

Accordingly, the future of VT management may evolve from ‘drug vs. ablation’ dichotomy towards a personalized substrate-based strategy. In this model, timing would no longer be determined solely by arrhythmia recurrence but also by substrate characteristics, clinical trajectory, expected tolerance to chronic AAD therapy, and overall disease phenotype.

The current data therefore suggest that the field is moving from rescue ablation towards preventive substrate management, although the optimal timing remains unresolved.

Ultimately, the question may no longer be simply ‘how early should we ablate VT?’, but rather ‘which patient should be treated early, based on which substrate, and in which centre?’.

Until then, the work by Façanha *et al*. provides contemporary evidence supporting a gradual but meaningful shift in VT management—from reactive rescue therapy towards earlier preventive intervention.

## Data Availability

No new data were generated or analysed in support of this research.
